# Overcoming Barriers: The AO Foundation’s Role in Latin American Scientific Growth

**DOI:** 10.3390/cmtr18010011

**Published:** 2025-02-05

**Authors:** Rodrigo dos Santos Pereira, Rafael Vago Cypriano, Carlos Gaete Garcia, Juan José Larrañaga, Nicolas Homsi

**Affiliations:** 1 Research & Development Commission—AOCMF Latin America, Rio de Janeiro 25961-260, Brazil; 2 Community Development—AOCMF Latin America, Vitória 29056-250, Brazil; rafaelcypriano@gmail.com; 3 Education Commission—AOCMF Latin America, Santiago 7910000, Chile; drgaete@gmail.com; 4 Regional Affairs—AOCMF Latin America, Buenos Aires C1198AAW, Argentina; juan.larranaga@hospitalitaliano.org.ar; 5 Regional Chair—AOCMF Latin America, Rio de Janeiro 22640-102, Brazil; nicolas@bucomaxilofacial.com

**Keywords:** Latin America, research, AO Foundation

## Abstract

This manuscript presents an exploratory evaluation of the challenges and opportunities in scientific research among craniomaxillofacial surgeons in Latin America. It focuses on initiatives introduced by the AO Foundation’s Research and Development (R&D) Committee to assess the current state of research involvement among AO Foundation members in the region and identify barriers to research. A survey conducted in 2023 among Latin American members of the AO Foundation gathered data on their interest in research, obstacles faced, and awareness of available opportunities, such as grants, fellowships, and mentorship programs. The outcomes revealed a strong interest in research, with 96.5% of respondents expressing a desire to engage. However, key barriers included limited time (46.5%), difficulties in project structuring and scientific writing (32.6%), and challenges in publishing (30.2%). Notably, 54.7% of respondents were unaware of the AO PEER program, and 65.6% were unfamiliar with the foundation’s research grant opportunities. The AO Foundation aims to enhance scientific development in Latin America by promoting multicenter research studies, training opportunities, and developing research group leaders. These strategies seek to support and encourage surgeons in advancing their scientific activities.

Building and developing science is not a paved road without detours where positive or negative results will be obtained. In low- and middle-income countries, the challenges are diverse, such as low wages, a lack of ideal infrastructure, and the absence of incentive policies and funding [1]. In addition to these limitations, Latin America (LA) faces a language barrier with other external cultures, particularly with English. In LA, Spanish and its variations predominate, with Brazil being the only country in the region using Portuguese. Access to journals and articles is limited, and when available, most studies are in English, creating a barrier for many students in the region. As previously mentioned, educational policies are limited, and the mandatory teaching of English is not part of these countries’ cultures, resulting in low access to scientific works for the population [2].

The AO Foundation has long been known in Latin America as a promoter of continuing education through the dissemination of its philosophy since the 1950s. Since then, it has been sustained through four pillars: education, documentation, instrumentation, and research. Research has been conducted since 1959 through the Experimental Surgery Laboratory, which today is known as the AO Research Institute Davos (ARI), developing advanced techniques and innovations in the treatment of the musculoskeletal system.

In 2023, the AO Foundation restructured the regional directorates into committees: Regional Affairs (RA), Education, Community Development (CD), and Research and Development (R&D), managed by a regional chair. This composition helped foster closer contact with members and improved communication. The new format improved responsibilities for each of the activities; nevertheless, the countries still have their representatives. However, many Latin American members were still unaware of the research opportunities offered by the foundation. In this context, the R&D committee conducted a survey in April 2024 among its members to identify regional aspirations and develop educational research policies.

The survey was sent by e-mail to 456 members using the website https://www.surveymonkey.com and answered by 86 (18.8%) from 13 different countries in the region; it included questions about the region’s research development and knowledge on the topic (Figure 1). Regarding surgeons’ interest in conducting research, 96.5% expressed interest, though 33.7% reported difficulties in development, 34.9% had little time, and 27.9% conducted research often (Figure 2). The main difficulties cited were time dedication (46.5%), structuring the project and scientific writing (32.6%), publishing work (30.2%), and defining the method (22.1%). Additionally, 18.6% had difficulties defining the study type, 14% had issues with the treatment of results, and 11.6% had issues obtaining the bibliography (Figure 3).

Regarding time availability for research, 48.8% reported having less than 4 h per week, 32.6% up to 4 h, 8.1% up to 8 h, and 10.5% more than 8 h (Figure 4). When asked about their number of publications (regardless of work type), 41.9% reported publishing fewer than 5 articles, 22.1% up to 10 articles, and 36% more than 10. Another question asked about their interest in taking part in a multicenter study in the region, with 95.3% responding positively. These data show participants have some experience in article development but limited dedication time.

In an editorial, Hupp [3] reported that the decrease in publications in the United States is due to fewer residents taking part in scientific studies. This is justified by the type of exclusively technical training provided by educational institutions, leaving no time for scientific development. Encouraging scientific projects is essential for training critical thinking on scientific evidence and academic article interpretation. Trindade [4] highlighted that dedication time in craniofacial surgery research in LA is a significant obstacle. However, he also noted the lack of clinical training, leadership in projects, duration, and financial incentives as barriers faced by regional scientists.

Despite LA’s extensive region with various countries, only Brazil, Chile, Argentina, and Mexico have international scientific recognition and influence [4]. These countries have more research development, reflected in collaborations with countries that have less research incentive. This is seen mainly in Brazil, where universities are encouraged to invest in the internationalization of their postgraduate programs, fostering both scientific and educational interaction [4].

One of the main regional problems is related to English, the common language of science [2]. For non-native readers, reading time increases, resulting in comprehension difficulties and interpretation errors [5]. Although Spanish-language journals exist, most are not indexed in major electronic databases (e.g., PubMed, Scopus, and the Web of Science), reducing article visibility [6]. Despite these challenges, AOCMF members show a willingness to face them but need support and guidance.

According to José-Abrego and Panduro [1], strengthening medical societies and foundations prepares surgeons to face medical practice challenges. The AO Foundation has played this educational role for over six decades, combining education, research, and financial support through funding. One of the foundation’s projects is the Program for Education and Excellence in Research (AO PEER); however, 34.9% of AOCMF-LAT members are unaware of its existence. Additionally, 43% have never taken part despite knowing about it (Figure 5). Another reported data point is that 65.6% of participants are unaware of the foundation’s research grant opportunities.

Franco [7] reported that dedicating time to research development can lead to technical and educational growth in the specialty. Furthermore, a work plan involves changing the traditional education system to a hybrid model with the advantage of virtual learning. In this context, the AO Foundation has shown satisfactory results through Study Clubs and AO PEER.

However, all changes require adaptation time. Hupp [8] argues that reflection is essential in forming critical thinking and learning from others’ experiences. In 2023, the AO Foundation chose Latin America as the first region to receive the new board structure. This allowed for more inclusion, diversity, and equity through decentralized decision-making, enabling each committee to work together on specific regional growth goals.

According to Alfaro-Toloza and Olmos-de-Aguilera [9], investing in junior surgeons has enormous potential to form scientific expertise in developing countries, playing a crucial role in encouraging and motivating future generations. In this regard, the AO Foundation supports young researchers financially through the Start-up Grant program, providing up to CHF 50,000 per year, access to major specialty journals through membership, and fellowships: Clinical Research Fellowship and AO Research Institute Davos Medical Research Fellowship.

With the development of Latin America in mind, in 2022, through the international CD, a mentorship incubator project was started for participants wishing to develop their projects. These participants already had their ideas and/or projects, resulting in applications solely from Brazil. Aiming to expand these mentorships and, with the survey results, the R&D and regional CD developed the Research Pathway program. This program is set to begin in the first half of 2025 and will consist of modules to help members develop their projects from the key question to the applied method. This initiative aims to promote education in scientific principles and provide mentorship. However, it is an engagement that seeks long-term rather than short-term results, recognizing the region’s potential for the competence of junior surgeons [10].

Thus, considering regional scientific development, the AO Foundation, through its board, aims to promote listed strategies to encourage LA surgeons to develop their scientific activities. These include setting up agreements through multicenter studies according to each institution’s feasibility and infrastructure, offering support and training to interested parties through funding applications and fellowships, and forming research group leaders in the medium and long term.

## Figures and Tables

**Figure 1:**
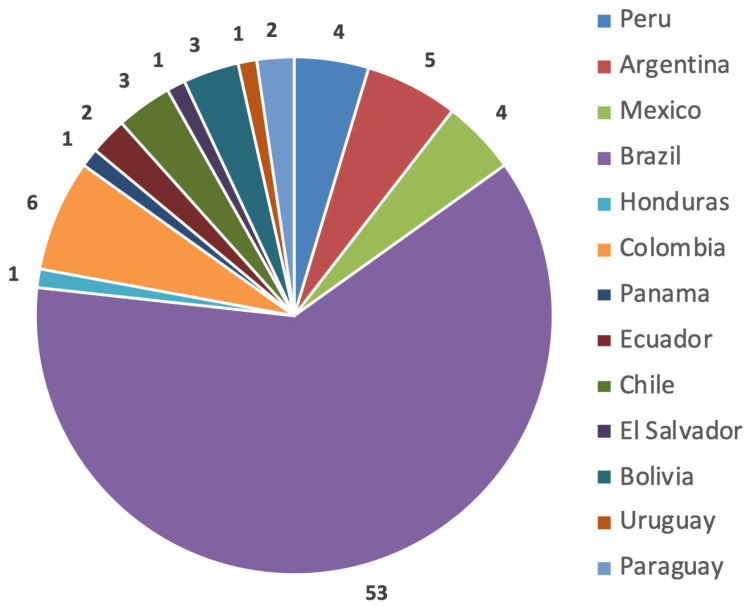
Graphic demonstrating the number of participants per country.

**Figure 2:**
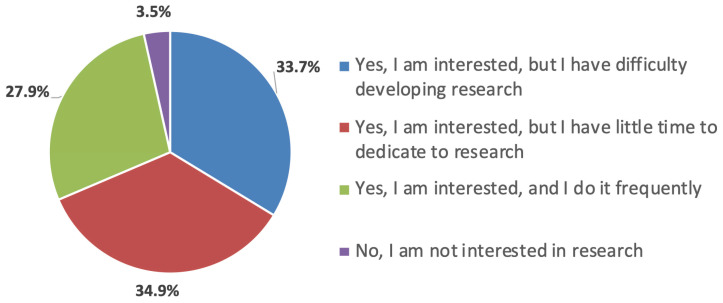
Graphic demonstrating the interest of the members in developing research.

**Figure 3:**
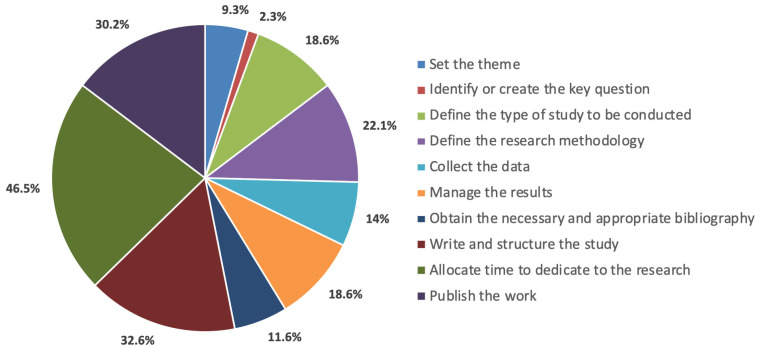
Graphic demonstrating the main difficulties of the members in developing research.

**Figure 4:**
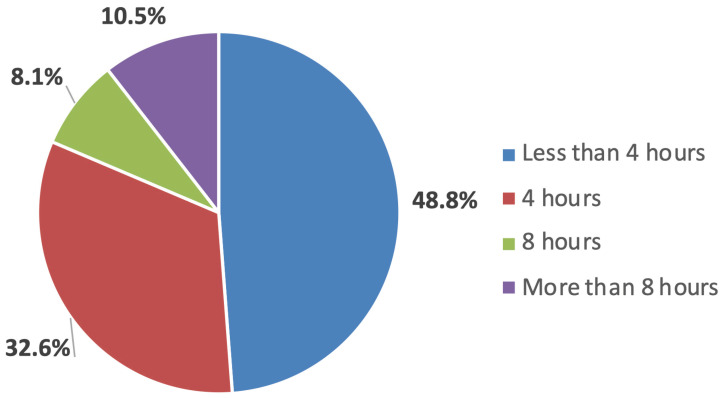
Graphic showing the availability in time of the members to conduct research.

**Figure 5:**
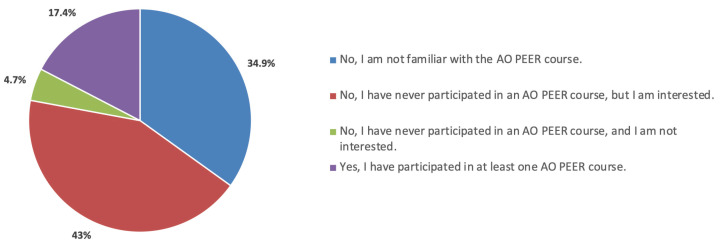
Graphic demonstrating interest of the members about AO PEER.

## Data Availability

No new data were created or analyzed in this study. Data sharing is not applicable to this article.
